# A case of culture‐negative endocarditis causing flail perforated mitral valve

**DOI:** 10.1002/ccr3.8556

**Published:** 2024-02-27

**Authors:** Sriharsha Dadana, Anusha Kondapalli, Vipul Madhwani

**Affiliations:** ^1^ Department of Hospital Medicine Cheyenne Regional Medical Center Cheyenne Wyoming USA; ^2^ Division of Cardiology Cheyenne Regional Medical Center Cheyenne Wyoming USA

**Keywords:** culture‐negative endocarditis, endocarditis, flail mitral valve, mitral regurgitation, mitral valve perforation

## Abstract

We describe a case of culture negative endocarditis causing mitral valve perforations and recurrent heart failure admissions.

## 1 CASE PRESENTATION

**FIGURE 1:**
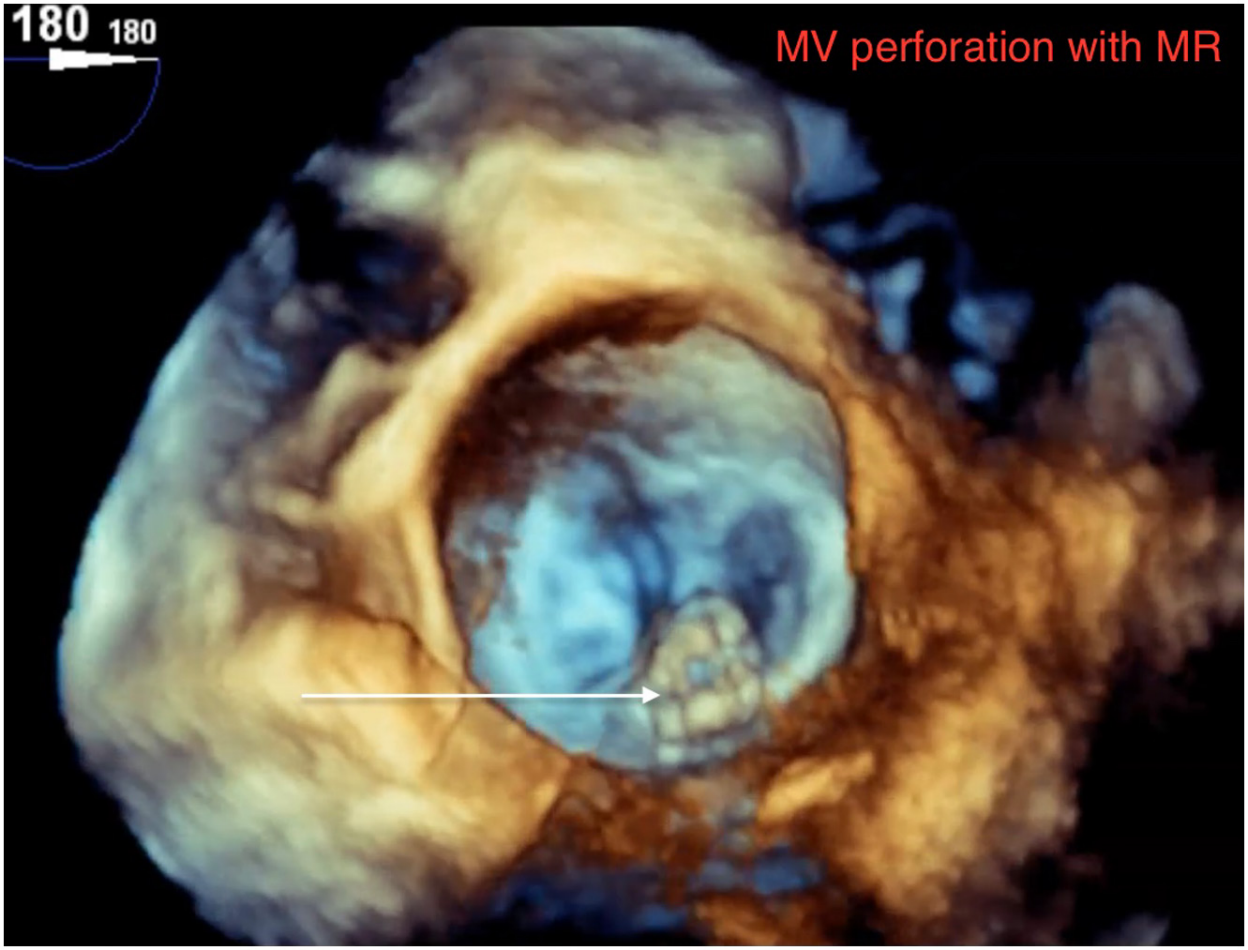
MV perforation with mitral regurgitation.

**FIGURE 2:**
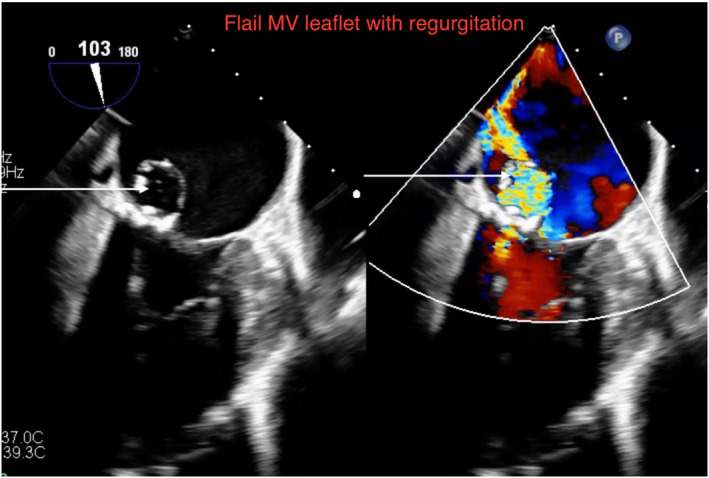
Echo showing flail MV leaflet with corresponding doppler showing regurgitation.

A 65‐year‐old female with recurrent heart failure exacerbations had echocardiogram showing ejection fraction 55%, moderate to severe mitral regurgitation. Transesophageal echo with doppler showed mitral valve has flail P2 scallop with perforation and moderate to severe eccentric regurgitation (Figures 1 and 2; Videos S1 and S2). Patient had urosepsis and *E. coli* bacteremia 6 months prior which likely was the source of valvular infection and perforation. She was deemed not a surgical candidate given comorbid illnesses. While early surgical valve replacement is associated with lower mortality, it is done in only half the cases due to prohibitive surgical risk.^1^, ^2^

## AUTHOR CONTRIBUTIONS

**Sriharsha Dadana:** Conceptualization; data curation; supervision; validation; visualization; writing – original draft; writing – review and editing. **Anusha Kondapalli:** Conceptualization; data curation; validation; visualization; writing – original draft; writing – review and editing. **Vipul Madhwani:** Supervision; validation; visualization.

## FUNDING INFORMATION

None.

## CONFLICT OF INTEREST STATEMENT

The authors declare no conflict of interest.

## CONSENT

Written informed consent was obtained from patient to publish this article in accordance with journal's patient consent policy.

## Supporting information


Video 1

Video 2

## Data Availability

All data underlying the results are available as part of the article, and no additional source data is required.
